# Heterogeneous Genetic Diversity Estimation of a Promising Domestication Medicinal Motherwort *Leonurus Cardiaca* Based on Chloroplast Genome Resources

**DOI:** 10.3389/fgene.2021.721022

**Published:** 2021-09-15

**Authors:** Jiahui Sun, Yiheng Wang, Thomas Avery Garran, Ping Qiao, Mengli Wang, Qingjun Yuan, Lanping Guo, Luqi Huang

**Affiliations:** ^1^ National Resource Center for Chinese Materia Medica, China Academy of Chinese Medical Sciences, Beijing, China; ^2^ Academician workstation, Jiangxi University of Traditional Chinese Medicine, Nanchang, China

**Keywords:** traditional herbal medicine, new medicinal source, simple sequence repeat, INDEL, codon usage, intraspecific variation

## Abstract

*Leonurus cardiaca* has a long history of use in western herbal medicine and is applied for the treatment of gynaecological conditions, anxiety, and heart diseases. Because of its botanical relationship to the primary Chinese species, *L. japonicus*, and extensive medical indications that go beyond the traditional indications for the Chinese species, it is a promising medicinal resource. Therefore, the features of genetic diversity and variability in the species have been prioritized. To explore these issues, we sequenced the chloroplast genomes of 22 accessions of *L. cardiaca* from different geographical locations worldwide using high-throughput sequencing. The results indicate that *L. cardiaca* has a typical quadripartite structure and range from 1,51,236 bp to 1,51,831 bp in size, forming eight haplotypes. The genomes all contain 114 distinct genes, including 80 protein-coding genes, 30 transfer RNA genes and four ribosomal RNA genes. Comparative analysis showed abundant diversity of single nucleotide polymorphisms (SNPs), indels, simple sequence repeats (SSRs) in 22 accessions. Codon usage showed highly similar results for *L. cardiaca* species. The phylogenetic and network analysis indicated 22 accessions forming four clades that were partly related to the geographical distribution. In summary, our study highlights the advantage of chloroplast genome with large data sets in intraspecific diversity evaluation and provides a new tool to facilitate medicinal plant conservation and domestication.

## Introduction

The *Leonurus* genus consists of herbaceous perennial plants that are widely distributed in Asia and Europe and naturalized in America and Africa (National Commission of Chinese Pharmacopoeia, 2015). One species that has long been used in Western herbal medicine and is commonly called “motherwort,” is *Leonurus cardiaca*. As the vernacular name indicates, it is applied for the treatment of gynaecological diseases. *L. cardiaca* is the typical species used medicinally of the *Leonurus* genus in Western countries, such as England, Poland, Bulgaria, Czech, the United States, etc. (Paweł et al., 2014; Upton, 2018). *L. cardiaca*, like its Chinese cousin, is frequently used for regulating menstruation and treating other gynecological diseases. However, it is also commonly used to treat anxiety, sleeplessness, and heart diseases (Shikov et al., 2011; Pitschmann et al., 2017; Zhang et al., 2018; Garran et al., 2019; Garran, 2020). And, unlike its Chinese cousin, it is a perennial, and because of the harvesting methods used, it can be harvested for several years without need for tilling the soil, which may be of benefit to conservation of soil and water. Due to its wide clinical use and economic value, *L. cardiaca* is a meritorious medicinal resource that hosts promising values for domestication and cultivation in China.

Typical of many domesticated medicinal plants, the domestication of the wild plants may reduce genetic diversity, which can result in a genetic bottleneck (Doebley et al., 2006; Miller and Gross, 2011; Wang et al., 2020). Although it is impossible to know how much of the original wild genetic diversity was brought into the progenitor population, it is likely that, at least, some genetic diversity has been left behind. At the same time, the domestication of the wild plants are under the effects of artificial selection periods to meet human needs, which likely led to a decrease of genetic diversity. Therefore, understanding the population structure and genetic diversity of *L. cardiaca* is an important step for more in-depth investigation in order to avoid potentially serious genetic problems in the future.

Genetic diversity is the basis of evolutionary change, including all types of variation of single nucleotide polymorphisms (SNPs), insertion-deletion (Indel), and structural variation (Sv). Deterministic and stochastic forces, over millions of years, including natural selection, adaptation, and genetic drift have created abundant genetic diversity that generates considerable genotypic and phenotypic diversity. It is also the foundation of species, population, and individual diversity (Nevo, 2001). For medicinal plants, by all means of molecular features, genetic diversity is crucially important for species identification and to guide breeding population selection. Effective introduction, conservation, and utilization of medicinal plants also requires clarity of genetic diversity. And, considering that many important medicinal plants are facing severe threats due to increasing demand, overharvesting, and habitat loss, the determination of genetic diversity is urgently needed for better introduction, conservation, and utilization of medicinal plants.

Previously, several biomarkers have been used to evaluate the genetic diversity of *L. cardiaca*, such as amplified fragment length polymorphism (AFLP), random amplified polymorphic DNA (RAPD), inter-simple sequence repeats (ISSR), inter-retrotransposon amplified polymorphism (IRAP), and the inter-primer binding site (iPBS) (Khadivi-Khub and Soorni, 2014; Borna et al., 2017), indicating that a number of molecular variations exist among *L. cardiaca* populations. However, some advanced biomarkers, such as the chloroplast genome, can provide much more information for genetic diversity studies.

Chloroplasts are key organelles for photosynthesis and other biochemical pathways in plants. A chloroplast has its own genome. Determination of chloroplast genomes was once carried out by either isolation of chloroplasts or PCR amplification of whole chloroplast genomes, both of which are laborious and time-consuming (Nock et al., 2011; Souza et al., 2019). With the advent of next-generation sequencing technologies, a chloroplast genome can be determined by sequencing total genomic DNA and *de novo* assembling whole chloroplast genomes (McPherson et al., 2013; Brozynska et al., 2014). The chloroplast genome usually has a typical circular quadripartite structure, including a small single copy region (SSC) and a large single copy region (LSC), which are separated by a pair of inverted repeat regions (IRa, IRb) and harbor 110 to 130 genes with sizes ranging from 120 to 165 kb (Ravi et al., 2008; Cheng et al., 2017). Exceptions have been found in Alismatales, Fabaceae, Geraniaceae, Aristolochiaceae, and many parasitic plants with abnormal genome sizes and structures (Csanad and Pal, 2014; Blazier et al., 2016; Ross et al., 2016; Sinn et al., 2018). Owing to rare recombination, small genome size, uniparental transmission, and moderate evolution rate, chloroplast genome research has been used extensively in different scientific fields. In molecular phylogeny, it can clearly reflect the relationship at different taxonomic levels, such as *Chaenomeles*, *Juglans*, *Coryloideae, Angelica,* and *Distylium,* and even difficult relationships within Fabaceae can be addressed (Dong et al., 2017; Hu et al., 2017; Dong et al., 2018; Hu et al., 2020; Sun et al., 2020; Zhang et al., 2020; Wang et al., 2021a; Dong et al., 2021)*.* In phylogeographical analysis, the advantage of the chloroplast genome in non-recombination and uniparental inheritance can allow for successfully estimation of divergence times and determine a biogeographic history (Zhang et al., 2017; Liu et al., 2018; Zhao et al., 2019; del Valle et al., 2019). Moreover, chloroplast genome markers, such as single nucleotide polymorphisms (SNPs) and simple sequence repeats (SSRs), can be used for population diversity estimations (Cao et al., 2018; Bautista et al., 2020; Ren et al., 2020).

Thus far, the chloroplast genome of *L. cardiaca* has not been reported. Since it is a new potential resource for domestication within China, we chose to use the chloroplast genome to infer the divergence within *L. cardiaca*. In this study, we sequenced 22 accessions of *L. cardiaca* chloroplast genomes from different geographical locations around the world using the Illumina HiSeq platform and compared heterogeneous divergence, such as SNPs, insertion/deletions, simple sequence repeats, and codon usage. The objectives of this study was 1) to determine the chloroplast genome of *L. cardiaca* and evaluate the intraspecific variation in this species and 2) to provide baseline data for genetic resources of *L. cardiaca,* including SNPs, SSRs, and indels, for genetic diversity assessment, to guide future domestication and conservation efforts.

## Materials and Methods

### Plant Material, DNA Extraction and Sequencing

A total of 22 *L. cardiaca* accessions were collected from China, United States, and Europe (Supplementary Table S1) to represent the distribution of this species. Voucher specimens were deposited at the herbarium of the Institute of Chinese Materia Medica (CMMI). Total genomic DNA was extracted from fresh leaves of a single individual using the Plant DNA Kit (D200-200, http://www.gene-better.cn/) from Genebetter Life Science Co., Ltd. and purified it using a Wizard DNA clean-up system (Promega, Madison, WI, United States). A paired-end library was constructed using a NEBNext UltraTM DNA library prep kit and PE150 sequencing of 22 accessions was conducted on an Illumina HiSeq XTen platform at Novogene (Tianjin, China).

### Genome Assembly and Annotation

Contigs were *de novo* assembled from the high-quality paired-end reads by using the SPAdes 3.6.1 program (Kmer = 95) (Bankevich et al., 2012) after low-quality reads were filtered using Trimmomatic 0.39 (Bolger et al., 2014). The chloroplast genome sequence contigs was extracted directly from the initial assembly by performing a BLAST search (Altschul et al., 1990) using the closely related species *L. japonicus* (GenBank: NC038062) as the reference. The selected contigs were assembled using Sequencher 5.4.5 (Gene Codes Corporation, Ann Arbor, MI United States, http://www.genecodes.com). A double-check process, Geneious 8.1, was used to map all reads to the assembled chloroplast genome sequence to verify the assembly accuracy (Kearse et al., 2012). The complete chloroplast genome sequences were annotated with Plann (Huang and Cronk, 2015) by using *L. japonicus* (GenBank: NC038062) as the reference and were manually adjusted in Sequin. The circle chloroplast map was drawn using the online program OrganellarGenomeDRAW (OGDRAW) (Lohse et al., 2007). The genome features of different regions were calculated using Geneious 8.1.

### Single Nucleotide Polymorphisms, Indels, and Divergent Hotspot Identification

A total of 22 chloroplast genomes were aligned using MAFFT online service in auto strategy (Katoh and Standley, 2013; Katoh et al., 2019) and manually adjusted by Se-al 2.0 (Rambaut, 2002); these sequences formed eight haplotypes. The SNPs and indels were calculated by DNasp (Librado and Rozas, 2009) and MEGA X (Kumar et al., 2018) in default parameter. The synonymous and nonsynonymous SNPs and nucleotide transitions and transversions were manually checked in MEGA. The nucleotide diversity of the chloroplast genome was calculated based on sliding window analysis using DnaSP v5.10 software with parameter settings of a 600-bp window length and 100-bp step length. The IR/SC boundary map of eight haplotypes was performed in IRscope (Amiryousefi et al., 2018).

### Codons and Microstructural Events

All protein-coding genes were isolated using a Python script written by Wuping (http://github.com/wpwupingwp/). The variable mutation sites, parsimony information sites, and relative synonymous codon usage (RSCU) values were analysed using DNasp and MEGA X. The heatmap from all RSCUs of the chloroplast genome was carried out using TBtools (Chen et al., 2020).

The software REPuter was used to visualize the dispersed and palindromic repeats with the following parameters: Hamming distance = 3, repeat size ≥30 bp and at least 90% similarity (Stefan et al., 2001). Tandem repeats were identified using the Tandem Repeats Finder with default parameters (Benson, 1999). Simple sequence repeats (SSRs) were obtained using Genome-wide Microsatellite Analysing Tool Package (GMATA) software (Wang and Wang, 2016) with the search parameters set at >10 repeat units for mononucleotides, >5 repeat units for dinucleotides, >4 repeat units for trinucleotides, >3 repeat units for tetranucleotide, pentanucleotide, and hexanucleotide SSRs. The sequence of “USOR-2” was used as a standard reference to count the microstructural events.

### Phylogenetic Inference and Network Analysis

The optimal substitution mode was finalized using ModelFinder (Kalyaanamoorthy et al., 2017). Phylogenetic analysis was carried out using RAxML v.8.2 in the maximum likelihood (ML) method with the GTR + G model (Stamatakis, 2014). Node support values were determined with 500 rapid bootstrap replicates; bootstrap values branches (<50%) were merged. Haplotype data was generated in DNasp and haplotype frequencies in populations were calculated by Arlequin v3.5.1.3 (Excoffier and Lischer, 2010), and a TCS network of 22 chloroplasts was generated by the network program PopArt v1.7 (Clement et al., 2000; Leigh and Bryant, 2015) using the haplotype data and populations haplotype frequencies data. *L. japonicus* (GenBank: NC038062) was used as an outgroup in both the phylogenetic analysis and TCS network.

## Result

### Characteristics of *L. cardiaca* Chloroplast Genome

The number of paired-end raw reads obtained by the Illumina HiSeq Xten system ranged from 16,330,634 to 31,010,620 for the 22 *L. cardiaca* accessions, clean reads of chloroplast genome ranging from 572,567 to 3,688,804 were extracted, yielding 566 × to 3,659 × chloroplast genome coverage (Supplementary Table S1). The accession numbers MZ274149 to MZ274170 of the complete chloroplast genome sequences were deposited in GenBank (Supplementary Table S1). The genome size ranged from 1,51,236 bp to 1,51,831 bp. All genome structures were extremely well conserved, and, as with most angiosperms, contained typical quadripartite structures with a pair of IR regions (25,644–25,653 bp), LSC regions (82,294–82,888 bp) and SSC regions (17,651–17,655 bp). The GC content of all the sequences was consistent in the LSC, SSC, and IR regions, accounting for 36.6, 32.2, and 43.4%, respectively. The main reason for the higher GC content of the IR regions was that the IR regions contained four high GC content rRNA genes. A total of 22 accessions of *L. cardiaca* forming eight haplotypes were obtained.

**FIGURE 1:**
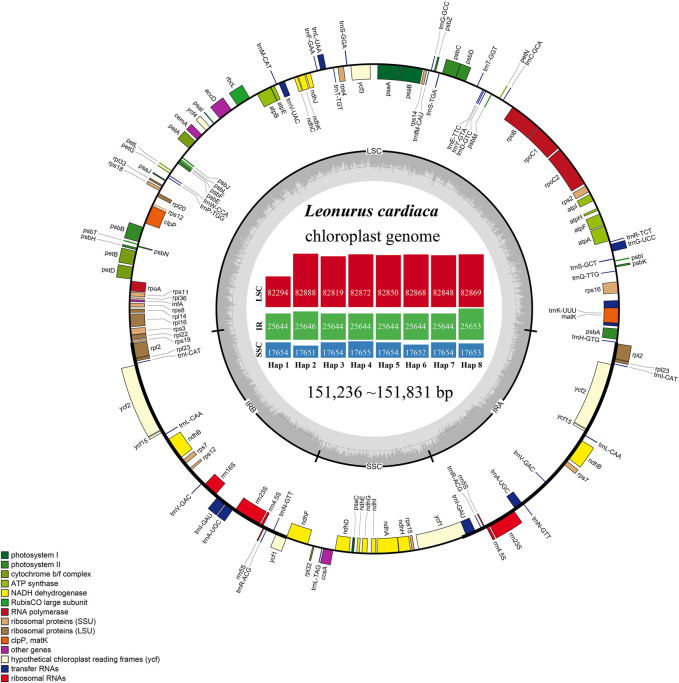
Gene maps of the chloroplast genomes of *L. cardiaca*. Genes on the inside of the large circle are transcribed clockwise and those on the outside are transcribed counter clockwise. The genes are color-coded based on their functions. The dashed area represents the GC composition of the chloroplast genome. The histogram inside represents different parts size of eight haplotypes.

*L. cardiaca* contained 114 distinct functional genes, including 80 protein-coding genes, 30 tRNA genes, and four rRNA genes. Eighteen genes were duplicated in the IR regions (Figure 1; Table 1). In addition, a total of 84 genes were located in LSC, including 62 protein-coding and 22 tRNA genes, while the SSC region harboured 11 protein-coding genes and one tRNA gene. Fourteen genes (*atpF*, *rpoC1*, *ndhB*, *petB*, *rpl2*, *ndhA*, *rps12*, *rps16*, *trnA*-*UGC*, *trnI*-*GAU*, *trnK*-*UUU*, *trnL*-*UAA*, *trnG*-*GCC* and *trnV*-*UAC*) contained a single intron, and two genes (*clpP* and *ycf3*) had two introns. The *trnK*-*UUU* gene had the largest intron, in which the *matK* gene was completely contained. The *rps12* gene is a trans-spliced gene with a 5′-end located in the LSC region and a 3′ end located in the IR region. The border regions and adjacent genes of eight haplotypes were compared to analyze the expansion and contraction in junction regions (Supplementary Figure S1). The expansion and contraction at the IR/SC borders exhibited completely identical structures among haplotypes. The IRb/LSC junction (JLB) occurred in the *rps19* gene, thus *rps19* had a 34 bp extension in the IRb region. The *ndhF* gene overlapped with the IRb/SSC junction (JSB) in 20 bp. The *ycf1* gene crossed over the IRa/SSC junction (JSA) in 1,084 bp, as well as in the JSB. In addition, IRa/LSC junction (JLA) extended into the *trnH-GUG* gene with only 1 bp.

**TABLE 1:** The basic chloroplast genome information of *L. cardiaca* species.

Category for genes	Group of genes	Name of genes
Photosynthesis related genes	Rubisco	*rbcL*
	PhotosystemⅠ	*psaA, psaB, psaC, psaI, psaJ*
	Assembly/stability of photosystem Ⅰ	*ycf3, ycf4
	Photosystem Ⅱ	*psbA, psbB, psbC, psbD, psbE, psbF, psbH, psbI, psbJ, psbK, psbL, psbM, psbN, psbT, psbZ*
	ATP synthase	atpA, atpB, atpE, *atpF, atpH, atpI
	Cytochrome b/f compelx	petA, *petB, *petD, petG, petL, petN
	Cytochrome c synthesis	*ccsA*
	NADPH dehydrogenase	*ndhA, *ndhB, ndhC, ndhD, ndhE, ndhF,ndhG, ndhH, ndhI, ndhJ, *ndhK*
Transcription and translation related genes	Transcription	rpoA, rpoB, *rpoC1, rpoC2
	Ribosomal proteins	rps2, rps3, rps4, rps7, rps8, rps11, *rps12, rps14,rps15, *rps16, rps18, rps19,*rpl2, rpl14, *rpl16, rpl20, rpl22, rpl23, rpl32, rpl33,rpl36
	Translation initiation factor	*infA*
RNA genes	Ribosomal RNA	*rrn5, rrn4.5, rrn16, rrn23*
	Transfer RNA	*trnA-UGC, trnC-GCA, trnD-GUC, trnE-UUC, trnF-GAA, *trnG-UCC, trnG-GCC, trnH-GUG, trnI-CAU, *trnI-GAU,*trnK-UUU, trnL-CAA, *trnL-UAA, trnL-UAG, trnfM-CAUI,trnM-CAU, trnN-GUU, trnP-UGG, trnQ-UUG,trnR-ACG, trnR-UCU, trnS-GCU, trnS-GGA, trnS-UGA, trnT-GGU,trnT-UGU, trnV-GAC, *trnV-UAC, trnW-CCA, trnY-GUA
Other genes	RNA processing	*matK*
	Carbon metabolism	*cemA*
	Fatty acid synthesis	*accD*
	Proteolysis	*clpP
Genes of unknown function	Conserved reading frames	*ycf1, ycf2, ycf15*

Intron-containing genes are marked by asterisks (*).

### Single Nucleotide Polymorphisms, Indels, and Divergent Hotspot Identification

**FIGURE 2:**
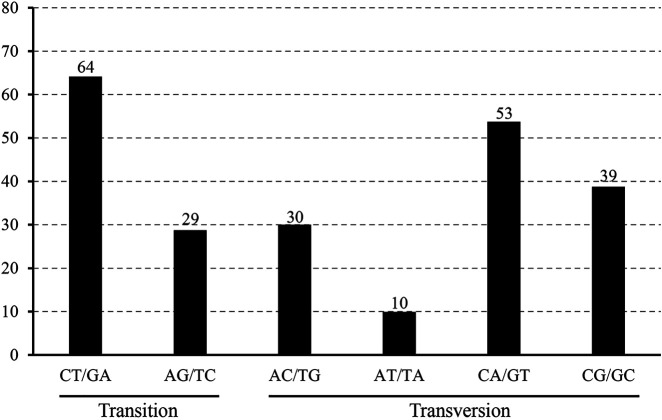
The patterns of nucleotide substitution among the eight *L. cardiaca* haplotypes. Nucleotide substitution were divided into six types as indicated by the six non-strand-specific base-substitution types (i.e., numbers of considered G to A and C to T sites for each respective set of associated mutation types).

The 22 alignment sequences were 1,52,010 bp in length and contained 225 SNP mutation sites (IR region only counted one time), including 83 singleton variable sites and 142 parsimony informative sites, forming eight haplotypes with a haplotype polymorphism of 0.732 (Table 2, Supplementary Table S1). The nucleotide diversity ranged from 0.00014 (IR region) to 0.00101 (SSC region) among the three different parts, and the overall nucleotide diversity was 0.00042. In the total 225 SNP mutations, 114 mutations were found in intergenic spacers with 99 located in exons and only 12 located in intron regions. The overall SNP density was 1.48 per kb (1.82 per kb in LSC, 3.62 per kb in SSC and 0.39 per kb) (Table 3). The 99 coding region SNPs were distributed in 34 different genes, meaning that some genes contained more than two SNPs, and 55 were nonsynonymous SNPs. Ten out of 34 genes contained more than 3 SNPs (*ycf1*, *rpoC2*, *ndhF*, *ndhH*, *matK*, *ndhD*, *psbA*, *ndhA*, *psaB* and *ycf2*) (Table 4). Among these ten highly SNP variable chloroplast coding genes, *ycf1* harboured a maximum of 21 SNPs, 17 were nonsynonymous SNPs and 4 synonymous SNPs, followed by nine SNPs in *rpoC2* and eight in *ndhF*. In addition, the highest SNP density of the coding genes occurred in *ndhH,* which contained 5.08 SNPs per kb. The amount and density of SNPs may indicate that for these 22 accessions, the coding genes *ycf1* and *ndhH* diverged markedly. The patterns of SNPs, 93 transitions (Ts) and 132 transversions (Tv) were counted, and the overall Ts:Tv rate was 0.705, indicating that it was in favor of transversions (Figure 2). The high frequency SNPs were C to T and G to A, and mutations from A to T and from T to A exhibited the lowest frequency.

**TABLE 2:** Haplotype diversity and mutation of 22 *L. cardiaca* accessions.

Location	Length	Polymorphic sites	Singleton variable sites	Parsimony informative sites	Nucleotide diversity	Number of hap	Hap diversity
LSC	83,040	151	60	91	0.00048	8	0.732
IR	25,654	10	3	7	0.00014	6	0.71
SSC	17,662	64	20	44	0.00101	6	0.71
Total	152,010	225	83	142	0.00042	8	0.732

**TABLE 3:** Summary of variants detected in *L. cardiaca* chloroplast genomes.

Variants	Exon	Spacer	Intron	Total	LSC density/kb	SSC density/kb	IR density/kb	Total density/kb
SNPs	99	114	12	225	1.82	3.62	0.39	1.48
Snon	55	—	—	—	—	—	—	—
Ssyn	44	—	—	—	—	—	—	—
Indels	3	39	7	49	0.48	0.28	0.16	0.32

**TABLE 4:** Highly single nucleotide polymorphism variable chloroplast protein coding genes of *L. cardiaca*.

Gene	Snps	Non n	Syn n	Length bp	Density of SNPs/kb	Gene products
*ycf1*	21	17	4	5,570	3.77	ATP-binding cassette glutathione S-conjugate transporter
*rpoC2*	9	5	4	4,191	2.15	DNA-directed RNA polymerase subunit beta''
*ndhF*	8	2	6	2,223	3.6	NAD(P)H-quinone oxidoreductase subunit 5
*ndhH*	6	3	3	1,182	5.08	NAD(P)H-quinone oxidoreductase subunit 7
*matK*	5	4	1	1,536	3.26	Tyrosine-protein kinase
*ndhD*	4	1	3	1,410	2.84	NAD(P)H-quinone oxidoreductase subunit 4
*psaA*	4	0	4	2,253	1.78	Photosystem I P700 chlorophyll a apoprotein A1
*ndhA*	3	2	1	1,095	2.74	NAD(P)H-quinone oxidoreductase subunit 1
*psaB*	3	0	3	2,205	1.36	Photosystem I P700 chlorophyll a apoprotein A2
*ycf2*	3	3	0	6,867	0.44	Unknown

The listed gene contain three or more SNPs

**FIGURE 3:**
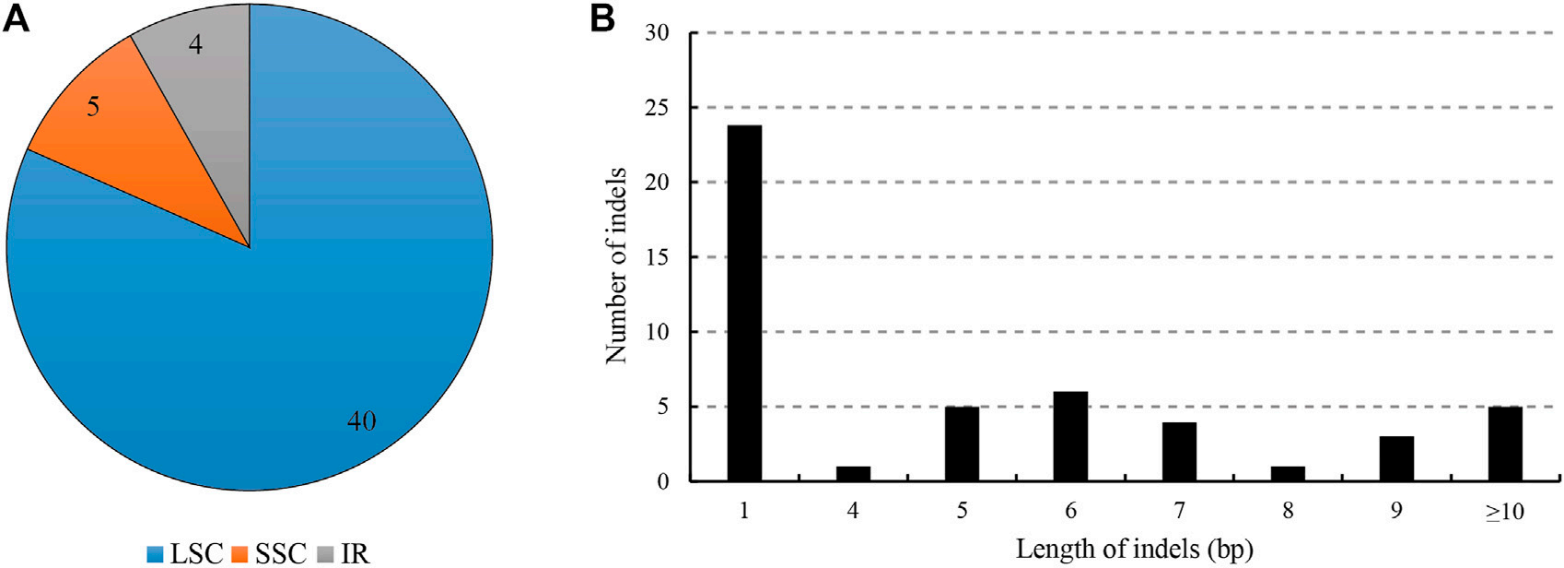
Analyses of indels in the *L. cardiaca* chloroplast genomes. **(A)** Count of indel types and locations. **(B)** Number and size of indels in chloroplast genomes.

For the indels, we retrieved 49 indels (IR region only counted one time), most of which occurred in the spacers (39), followed by the introns (7), and only three in the exons (40 in LSC, four in IR regions and five in SSC); the overall indel density was 0.32 per kb (Figure 3; Table 3). The three indels of the exons were located in *matK*, *rpoC1* and *trnV(UAC)*. The spacer *ndhF*-*rpl32* contained the highest number of indels (four), followed by *rbcL*-*accD* and *trnT* (*GGU*)-*psbD* (both three). The size of the indels ranged from one to 546 bp, and one base indel was most common. The largest indel was a deletion that occurred in the *trnC* (*GCA*)-*petN* of Hap1, and the second largest indel was a 52-bp insertion that occurred in the *petN*-*psbM* of Hap 2.

**FIGURE 4:**
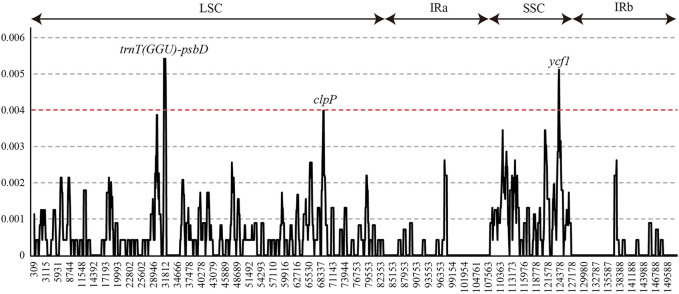
Sliding-window analysis of the whole chloroplast genomes of all *L. cardiaca* accessions.

Nucleotide diversity (Pi, π) was measured by DNAsp to identify the diversity hotspot regions among 22 *L. cardiaca* accessions in the whole chloroplast genomes (Figure 4). The Pi varied from 0 to 0.0054, while the average Pi was extremely low at only approximately 0.0005. Only three regions exceeded 0.04. The spacer of *trnT* (*GGU*)-*psbD* harbored the highest Pi values (Pi = 0.0054), followed by *ycf1* (Pi = 0.0051, most mutation located in the exon) and *clpP* (Pi = 0.0040, most mutation located in intron).

### Codon Usages

**FIGURE 5:**
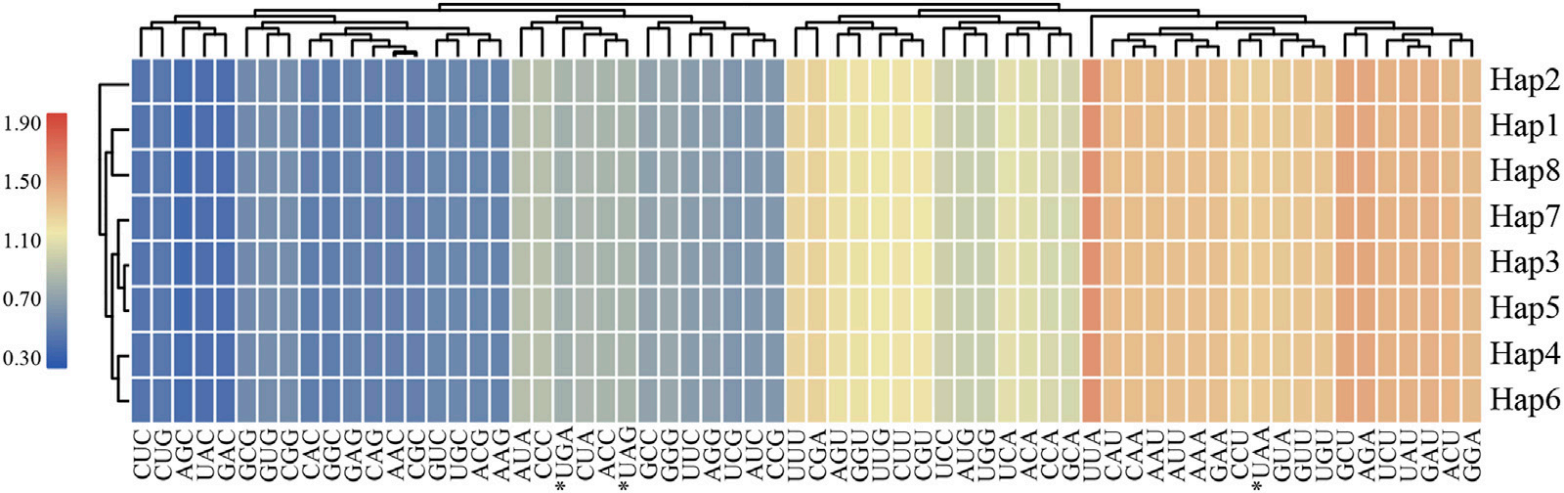
The RSCU values of all merged protein-coding genes for eight *L. cardiaca* haplotypes. Color key: the red values indicate higher values and the blue values indicate lower values.

In this study, eight haplotypes of protein-coding genes were used for further analysis in codon usage and relative synonymous codon usage (RSCU) were 68,508 ∼ 68,535 bp in length and encoded 22,836∼22,845 codons (Supplementary Table S2). Codon usage showed highly similar results for *L. cardiaca*. Among these codons, isoleucine was the most abundant amino acid encoded by the codons, ranging from 971 to 972 counts, whereas the stop codon encoded by “UAG” was the least abundant with only 20 counts. The RSCU values formed a heatmap, as shown in Figure 5. Red represents higher RSCU values, and blue indicates lower RSCU values. The RSCU values inferred that codons AGC and UUA represented the lowest and highest RSCU values, respectively, and codons AUG and UGG had no bias (RSCU = 1). Concurrently, the number of codons was equal in both higher (RSCU > 1) and lower (RSCU < 1) parts. In 31 higher frequency codons, the codons all ended with purine A or U, except UUG. Moreover, for all codons, a bias in favor of purine at the third codon position was apparent, as reported by other previous studies.

### Repeat Sequences and Simple Sequence Repeats

**FIGURE 6:**
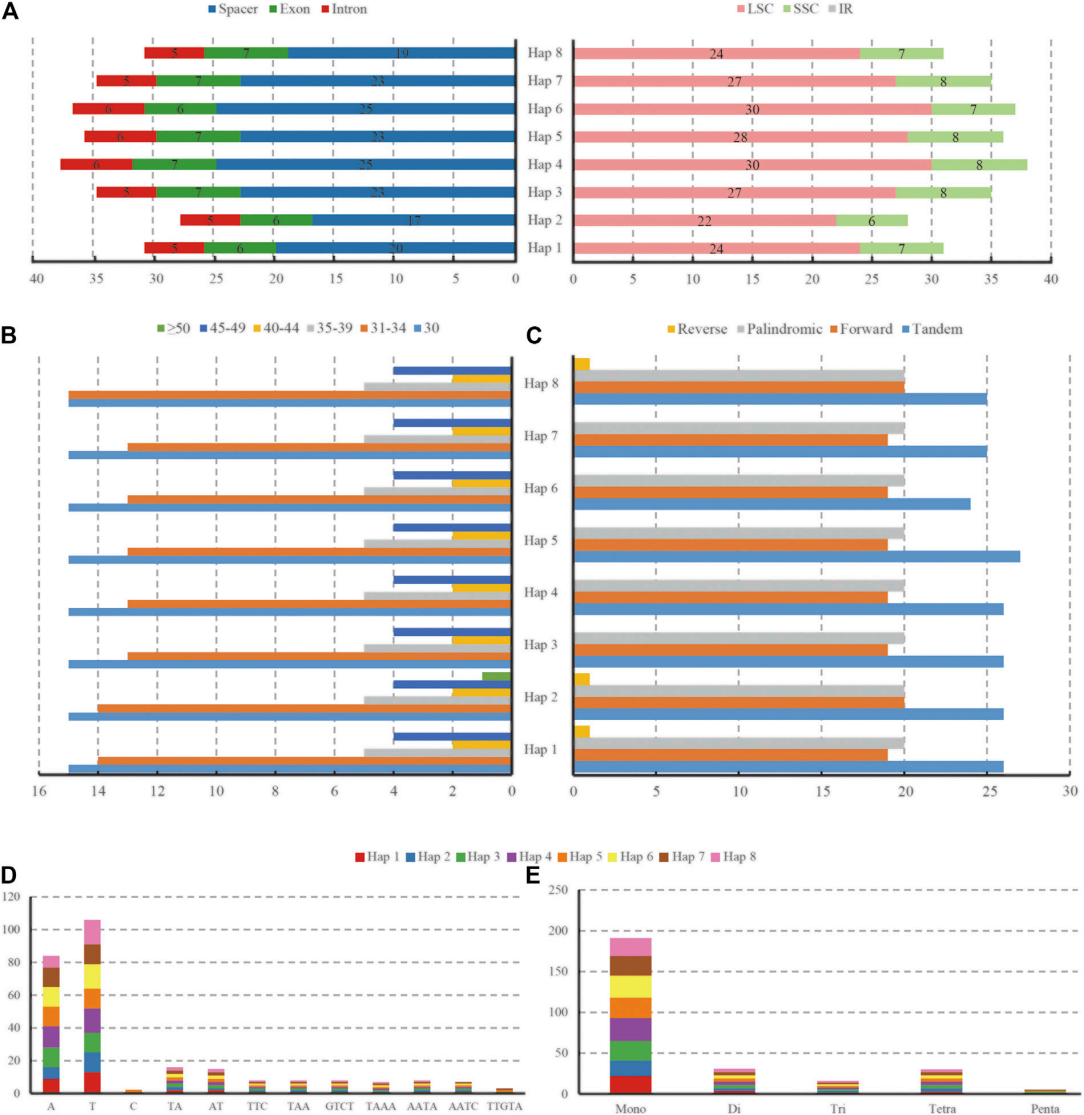
The type and distribution of SSRs in the eight haplotypes of *L. cardiaca* chloroplast genomes. **(A)** Number of SSR occurrence in different regions (LSC, SSC, IR and spacer, exon, intron). **(B)** Number of repeat sequences by length **(C)** Number of four repeat types **(D)** Number of identified SSR motifs in different repeat class types. **(E)** Number of SSR repeat types detected by GMATA.

We detected a total of 317 repeats with lengths of 30∼52 bp in the forward, palindromic, and reverse regions of eight haplotypes and 39∼41 repeats in each haplotype (Figure 6, Supplementary Table S3). Specifically, the number of forward repeats ranged from 19 to 20, which was slightly less than that of palindromic repeats (20), and only one reverse repeat existed in Haps 1, 2, and 8. However, the complementary repeat was absent in any of the haplotypes. According to the range of length, we classified the repeats into six groups, as shown in Figures 6B,C. The most common repeat was 30 bp, and 84.5% of repeats were limited to 30∼39 bp. Furthermore, 24∼27 tandem repeats were detected in eight haplotypes. In addition, a total of 271 SSRs, mono-, di-, tri-, tetra-, and penta-, were detected by GMATA analysis. The number of SSRs ranged from 28 (Hap 2) to 38 (Hap 4). Among these SSRs, there were 175 in the spacers, 53 in the exons, and 43 in the introns (212 in LSC and 59 in SSC, but none in IR regions) (Figure 6A). The majority of SSRs were mononucleotide repeats (70.5%), and most of them were A or T repeats (19–28). The dinucleotides and tetranucleotides were almost equal at 11.4 and 11.1%, respectively. The lowest numbers of repeat trinucleotides and pentanucleotides were 5.9 and 1.1%, respectively (Figures 6D,E).

Among all the haplotypes, 22 SSR loci showed polymorphisms after *in silico* analysis, 14 in spacers, 7 in introns, and only one was located in exons. The intron of *atpF* contained three polymorphic loci; the most compared with the others (Table 5).

**TABLE 5:** SSRs identified from in silico comparative analysis of the chloroplast genomes of eight haplotypes.

Region	Position	Location	Forward sequence	Reverse sequence	SSR type	Product size
LSC	*matK-trnK(UUU)*	Spacer	CCG​AAT​CGC​TCT​TTT​GAT​TTC	CCC​GAT​CTA​ACT​AGA​CGT​T	(A)11	175
LSC	*trnK(UUU)-rps16*	Spacer	AAGGATGTATAGATACAA	ATA​TTG​ACA​ACA​GTG​TAT​TG	(A)13	162
LSC	*rps16*	Intron	GAT​CAT​AAA​AGC​CCA​CCC​TT	TGGGCCCATTTGTCATTT	(C)10	206
LSC	*psbK-psbI*	Spacer	CTA​TAG​GGT​CTC​CCA​CAA​TA	ACT​ACG​GTA​TAA​ACG​AAG​AG	(T)10	199
LSC	*psbI-trnS(GCU)*	Spacer	GGC​GAA​ATC​CTG​GAC​GTG​AAG​AA	AAT​TGG​GAG​AGA​TGG​CTG​AGT​GGA	(A)10	220
LSC	*trnG(UCC)-trnR(UCU)*	Spacer	TTC​GAT​TCC​CGC​TAC​CCG​CTT​ATT	CGT​CCA​ATA​GGA​TTT​GAA​CCT​ATA​C	(T)10	242
LSC	*atpF*	Intron	CGA​TTC​ATT​TGG​CTC​TCG​CGC​TC	CTC​GAT​ATA​GAA​CAC​TCA​TCT​C	(T)10	296
LSC	*atpF*	Intron	ATT​TGG​CTC​TCG​CGC​TCA​A	GTC​AGA​AGA​GTC​CTC​TAA​GTA	(A)11	122
LSC	*atpF*	Intron	GAA​TGT​AAT​GAG​CCT​ATC​CTC​TTC	GGC​ATA​GGT​CGT​CGA​TTC​ATC​ATT​G	(A)10	170
LSC	*atpF-atpH*	Spacer	AAC​TCC​CGG​CAG​ATG​GCC​AGT​GGC​CCA​A	CCT​ACG​GGA​AAG​GCT​GAT​TCG​C	(T)14	285
LSC	*petN-psbM*	Spacer	GAA​TTT​GGA​ATC​TCA​TTG​G	GGT​CCG​TCT​TCC​TCA​GTA​TCC​ATC	(TTGTA)3	217
LSC	*ycf3*	Intron	CCA​GAA​CCT​CTA​GGT​GTA​ACC	TGC​TGG​CTA​TGC​CTA​GAT​CGC​GAA	(A)11	329
LSC	*trnF(GAA)-ndhJ*	Spacer	GGC​ACA​TGG​TTA​AAT​TGG​ATG​AGG	GTA​TCT​GAG​CCG​AAT​CTT​G	(TAAA)3	301
LSC	*ndhC-trnV(UAC)*	Spacer	CAC​CTA​GGA​TTC​TTG​CTA​AAG	CCT​AGC​CAA​GCA​CAC​CAA​AAC	(T)11	317
LSC	*petA-psbJ*	Spacer	CGGGTTTCCAGGTGTATC	CAC​CTC​TTC​CTC​GAT​CTT​G	(AT)5	268
LSC	*rpl33-rps18*	Spacer	GTA​TGT​CTT​ATC​CTT​GAA​AGG​GG	CGC​CTA​CGA​AAA​GAT​CGC​TTG​G	(A)12	170
LSC	*clpP*	Intron	CGC​TGA​ACT​GGA​CTT​CCG​A	ATT​CCC​TCA​CGC​TTG​GCG​CCA	(A)10	205
LSC	*petB*	intron	GGT​AGT​ATC​ACC​ACA​AAG​AAG​AG	TGA​GAT​AGG​CGA​AAA​CCC​CC	(T)16	193
LSC	*rpl14-rps3*	spacer	GCA​ATT​AAT​TTG​AAT​AGA​TAT​T	GAG​AAA​TGA​CGA​ACA​GGT​GCT	(A)10	180
SSC	*ndhF*	exon	AGC​CCA​CAT​ACG​ATG​AAG​AT	ATT​TGA​AGT​ATT​TAG​CAG​A	(A)11	281
SSC	*rpl32-trnL(UAG)*	spacer	TTA​TCA​TTT​GCA​AGG​CGG​G	CTCTTGTAAATCCTAC	(T)11	336
SSC	*rps15-ycf1*	Spacer	CAGATATGGATTTTACCG	TTC​GAT​CTC​GAA​ATG​AAT​CAC​C	(AATC)3	207

### Interspecific Relationships According to Phylogenetic and Network Analysis

**FIGURE 7:**
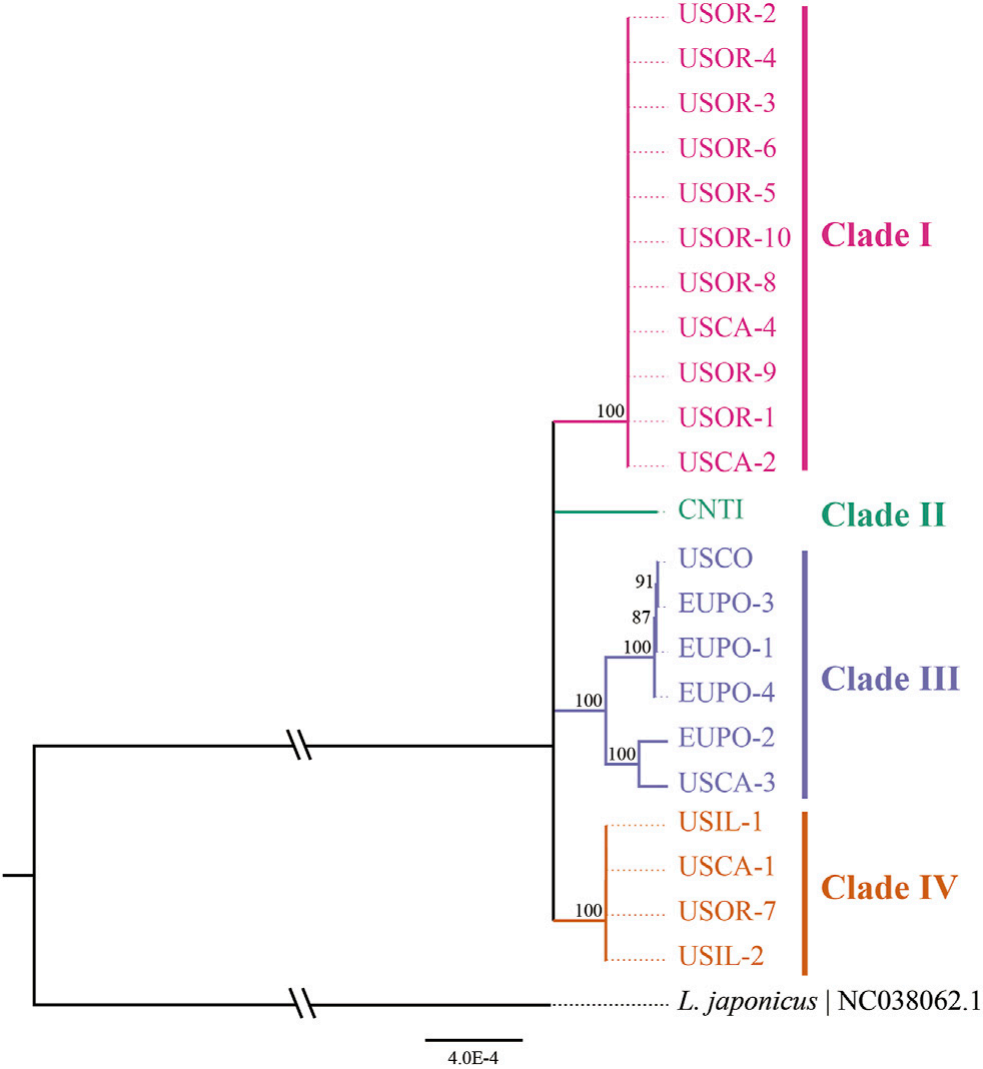
Phylogenetic relationships among 22 *L. cardiaca* accessions constructed from complete chloroplast genome sequences using maximum likelihood (ML). The ML topology is shown, with the ML bootstrap support value for each node. 22 accessions cluster to four clades show by different color.

**FIGURE 8:**
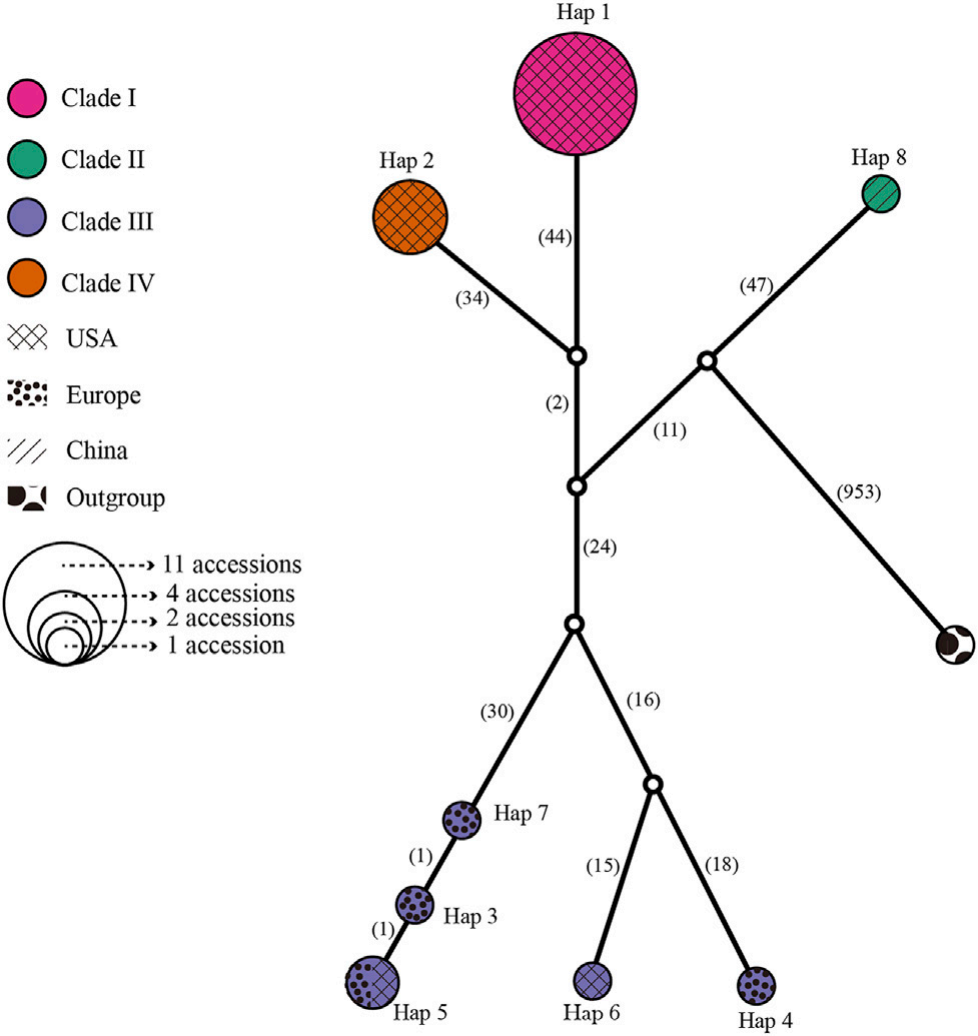
Network for all 22 *L. cardiaca* accessions base on chloroplast genome sequences. The relative size of each circle corresponds to proportional to haplotype frequencies. Different color represented different clades consistent with phylogenetic relationship. Different texture represented different distributions.

A median-joining network and phylogenetic analysis were carried out based on the 22 entire chloroplast genomes of *L. cardiaca*. The ML analysis strongly indicated significant divergence among 22 accessions, forming four clades with essential bootstrap support. In addition, samples from Tibet formed a clade separately, and clades I and IV were all from samples collected in the United States. However, clade III contained samples from different distributions from the United States and Europe (Figure 7). Regardless, the network result was largely comparable to the phylogenetic result. Additionally, four clades clustered by eight haplotypes, while Hap 1 contained 11 accessions, Hap 2 contained four accessions, and Hap 5 contained two accessions. The steps among haplotypes showed strong intraspecific variations (Figure 8).

## Discussion

### Conserved Chloroplast Genome Structure of *L. cardiaca*

The chloroplast genomes of *L. cardiaca* were determined for the first time in this study. Among the 22 chloroplast genomes, the sizes range from 1,51,236 bp to 1,51,831 bp, forming eight haplotypes. The structure of chloroplast genomes was highly conserved in *L. cardiaca*, gene order and contents, while no large genome rearrangement or gene loss was detected. All genomes harbor 114 distinct genes, including 80 protein-coding genes, 30 tRNA genes, and four rRNA genes, which is consistent with other related species (Lukas and Novak, 2013; Qian et al., 2013; Cheon et al., 2018). The genes *accD*, *rpl32*, and *ycf2* may be absent in some species, and they also do not occur in *L. cardiaca* (Jansen et al., 2007; Oliver et al., 2010; Wicke et al., 2011). The total GC content from different distributions is highly consistent, while the genome size varied slightly but not significantly. In addition, variations, such as GC content, gene content, and genome size, also has the potential to enable different species/populations or even individuals to be distinguished. All wild and cultivated accessions share the same quadripartite structure, gene number, order and IR/SC junction, and have a similar chloroplast size. Collectively, there is a highly conserved structure in the *L. cardiaca* chloroplast genome.

### Sequence Variability and Candidate DNA Barcodes of *L. cardiaca*

A total of 225 SNPs were detected in the 22 chloroplast genomes of *L. cardiaca*. According to the base frequency, the SNP mutations are likely to be from C to T or G to A. Transversion from A to T or from T to A is very rare. Most SNPs are located in LSCs, followed by SSC and IR regions, as are other mutation types, such as SSRs and indels. This is highly relevant to the region size and sequence conservation level. The number of SNPs of *L. cardiaca* (225 SNPs in 22 accessions) is relatively high when compared with that of other species, such as *Jacobaea vulgaris* (32 SNPs in 17 accessions), *Brassica napus* (294 SNPs in 488 accessions), *Brachypodium distachyon* (298 SNPs in 53 accessions) and *Macadamia integrifolia* (407 SNPs in 63 accessions) (Leonie et al., 2011; Qiao et al., 2016; Sancho et al., 2017; Nock et al., 2019). With respect to LSC, SSC, and IR regions, the SSC region is more variable with 3.62 SNPs per kb than LSC with 1.82 SNPs per kb. This result is consistent with other studies such as *Ricinus communis* and *Macadamia integrifolia* (Nock et al., 2019; Muraguri et al., 2020)*.* Due to evolutionary constrains and natural selection pressure, the coding regions are more conserved than the noncoding regions (Liu et al., 2019; Bautista et al., 2020; Zhang et al., 2021). The number of SNPs occurring in noncoding regions (126 SNPs) was greater than that in coding regions (99 SNPs). Among coding genes, the occurrence of nonsynonymous SNPs is more abundant than that of synonymous SNPs. As expected, this result can be deduced from the substitution pattern of SNPs because transversion tends to generate more nonsynonymous mutations. The *ycf1* and *ndhH* genes have much more SNPs than other genes. Approximately 80% of SNPs of *ycf1* are nonsynonymous and may cause the functional differentiation of *ycf1* across *L. cardiaca* populations worldwide. The *ycf1* gene could serve as a DNA barcode for *Leonurus* considering its better performance than the core plant barcodes matK and rbcL. This gene has been proposed as a candidate DNA barcode of angiosperms and is frequently applied in many species for identification and phylogenetic analysis (Dong et al., 2015). The *ndhH* gene is regarded as the best-performing marker in discrimination of grasses (Krawczyk et al., 2018). The noncoding regions usually have higher sequence variability compared to coding regions. The spacer of *trnT*-*psbD* and intron of *clpP* are two mutation hotspots though they are not as variable as *ycf1*. The spacer of *trnT*-*psbD* has been considered an effective locus for phylogenetic research at low taxonomic levels (Dong et al., 2012). Therefore, these four loci could be used as candidate DNA barcodes for species authentication in the *Leonurus* genus.

### Chloroplast Genome Structural Variations and Features of Codon Usage

Genome structural mutations are yet another form of information useful in revealing genetic diversity of species, population biology, or evolution. SSRs are the most common structural mutations, which cause insertions or deletions (McDonald et al., 2011; Ahmed et al., 2012; Yi et al., 2013; Abdullah et al., 2021). The most common SSRs in *L. cardiaca* chloroplast genomes are mononucleotides mainly composed of A or T and rarely G or C. There are significantly fewer Di-, Tetra-, Tri-, and Penta-nuclotide motif repeats. This phenomenon is similar to that of other studies in *Atractylodes* and *Chaenomeles* (Ahmed et al., 2012; Sinn et al., 2018; Hu et al., 2020; Sun et al., 2020; Wang et al., 2021b). The SSR primers designed in this study for amplifying the 22 polymorphic loci are expected to facilitate evaluation of genetic diversity of *L. cardiaca*. There are two unique patterns in the relative synonymous codon usage (RSCU) and usage frequency based on eight haplotypes of protein-coding genes. First, all the high-frequency codons have a bias in favor of ending with A or U, but in low-frequency codons, they are biased towards G or C at the third codon position. Second, two start codons (UGG and AUG) have no bias yet. This also agrees with previous studies (Meng et al., 2018; Bautista et al., 2020; Ren et al., 2020).

Collectively, except for codon usage, SNPs, indels and SSRs vary within the *L. cardiaca* accessions and could be qualified genetic resources to evaluate the diversity among populations. Compared with other species as mentioned above (*Jacobaea vulgaris*, *Brassica napus*, etc.), *L. cardiaca* showed higher genetic diversity.

### Tracing the Original Sources of Introduced *L. cardiaca*

The 22 genomes belong to eight haplotypes. These haplotypes form six clades. EUPO-1 (Poland, Hap 3) is a one-step mutant of EUPO-4 (Poland, Hap 7) and USCO (Colorado, Hap 5) is a one-step mutant of EUPO-1. *L. cardiaca* was naturalized from Europe to the United States. The nearly identical chloroplast genomes between EUPO-2 (Poland, Hap 4) and USCA-3 (California, Hap 6) and the Hap 5 containing accessions from Poland and Colorado could infer recent exchanges existed between the two continents. Four accessions from Europe contain their own unique haplotypes, indicating where the biodiversity center is. Close relationships among the genomes in Clade I and Clade IV indicate that the germplasm resources of *L. cardiaca* in United States have not been severely mixed. It is probably because *L. cardiaca* is not a wildly popular herb and very limited seed exchange has occurred (Yuan et al., 2010; Wang et al., 2020). Due to very limited samples available from Europe, it is impossible to locate the places of origin of most samples, unlike the one collected in Tibet. Apparently, the chloroplast genome information does provide an opportunity to trace the origins of introduced or invasive plants.

### Implication for Conservation of *L. cardiaca*

With the increasing demand for medical use, wild *L. cardiaca* resources are likely face over-exploitation. Bringing plants into cultivation as a crop is one solution. For the purpose of high yield and good quality, a seed bank for *L. cardiaca* needs to be constructed for easy access of the genetic resources during cultivar breeding. The genetic diversity revealed by the chloroplast genome information or the DNA barcodes proposed in this study could be used to guide the sampling processes and the subsequent evaluation of genetic integrity of this medicinal plant as was discussed by Yuan et al. (2010) for the Chinese medicinal plant *Scutellaria baicalensis* (Yuan et al., 2010).

In the period of seed bank construction, the chloroplast markers should be used to evaluate the overall genetic diversity and genetic structure of *L. cardiaca*. As has been shown in this study, the genetic diversity of *L. cardiaca* is highly structured and the seed bank to be built should contain as many variants as possible. During the maintenance stage of the seed bank, the molecular markers should be used to monitor any loss of genetic diversity due to founder effect, genetic drift, stochastic event, or other factors.

## Conclusion

Unambiguous genetic diversity and variability are not only prerequisites for the discovery of new medicinal resources but also a foundation of germplasm resource conservation and innovation. The genetic diversity of *L. cardiaca* was revealed with 22 chloroplast genomes. The biodiversity center is obviously in Europe because all four accessions have their own haplotypes. The molecular markers developed in this study could be used to guide the construction of germplasm resource banks either for conservation or cultivar breeding of this common medicinal plant. More extensive sampling in Europe is necessary for a better understanding of the overall genetic diversity and genetic structures of this species.

## Data Availability

The datasets presented in this study can be found in online repositories. The names of the repository/repositories and accession number(s) can be found below: Genbank (https://www.ncbi.nlm.nih.gov/), accession number (MZ274149 - MZ274170).
