# Supplementary figures and images for: Heterogeneous Genetic Diversity Estimation of a Promising Domestication Medicinal Motherwort Leonurus Cardiaca Based on Chloroplast Genome Resources
Source: Front Genet. 2021 Sep 15;12:721022. doi: 10.3389/fgene.2021.721022 (PMC8479170; doi:10.3389/fgene.2021.721022)

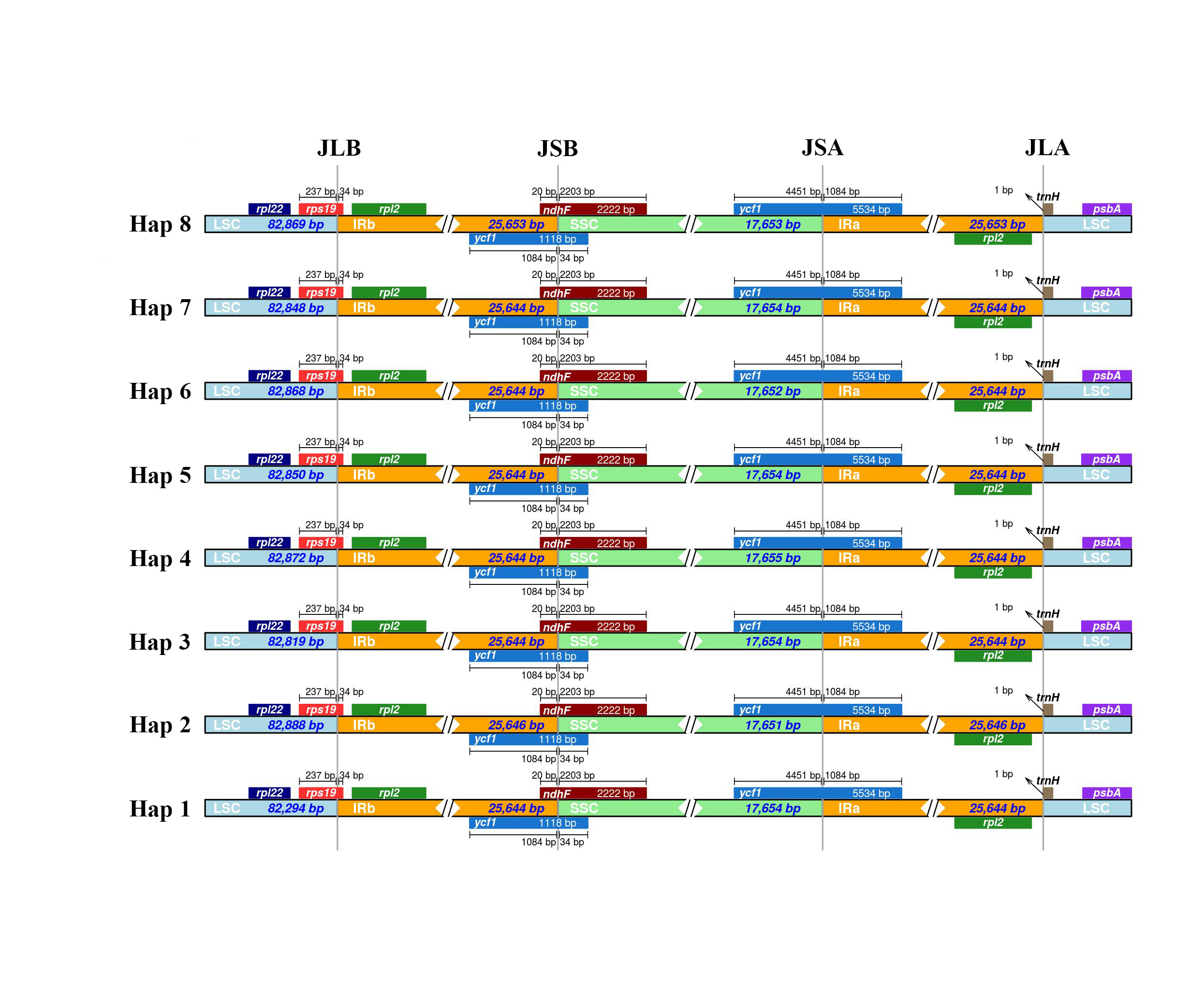

Supplement: Supplementary file 3 [file image1.jpeg]
